# Efficient upconversion luminescence from Ba_5_Gd_8_Zn_4_O_21_:Yb^3+^, Er^3+^ based on a demonstrated cross-relaxation process

**DOI:** 10.1038/srep22545

**Published:** 2016-03-02

**Authors:** Chao Mi, Jianhong Wu, Yanmin Yang, Boning Han, Jun Wei

**Affiliations:** 1 The Midwest Universities Comprehensive Strength Promotion Project, Hebei Key Lab of Optic-Electronic Information and Materials, College of Physics Science and Technology, Hebei University, Baoding 071002, China

## Abstract

Under 971 nm excitation, bright green and red emissions from Yb^3+^/Er^3+^ co-doped Ba_5_Gd_8_Zn_4_O_21_ phosphor can be observed, especially the intense red emission in highly doped samples. The experimental results indicate that Ba_5_Gd_8_Zn_4_O_21_:Yb^3+^, Er^3+^ emits stronger upconversion luminescence than NaYF_4_:Yb^3+^, Er^3+^ under a low excitation power, and a maximum upconversion power efficiency of 2.7% for Ba_5_Gd_8_Zn_4_O_21_:Yb^3+^, Er^3+^ was achieved. More significantly, to explain the red emission enhanced with the dopant concentration, this paper presents a possible cross-relaxation process and demonstrates it based on the rate equation description and temporal evolution. In view of the strong upconversion luminescence, colour tunable ability and stable chemical nature, Yb^3+^/Er^3+^ co-doped Ba_5_Gd_8_Zn_4_O_21_ phosphor could be an excellent candidate for efficient upconversion luminescence generation.

Upconversion (UC) is a nonlinear optical process characterized by the successive absorption of multiple pump photons via metastable states, followed by the emission of the luminescence at a shorter wavelength than the excitation wavelength^1^. In view of the unique optical properties, UC luminescence (UL) materials doped with rare earth (RE) ions have been utilized in potential applications, such as biological diagnosis, data storage, solar cells, solid display technique, solid-state lasers, and sensor technology^2^,^3^,^4^,^5^,^6^,^7^,^8^,^9^,^10^,^11^,^12^. However, one outstanding roadblock still exists: most of the UL materials fail to produce strong optical emission under low excitation power, which greatly limits the practical application in different fields. For example, owing to the weak emission signal, which limits the penetration depth to a few centimeters in bio-labeling and bio-imaging, UC luminescence bio-imaging could be used to obtain anatomical and physiological details only in small animals. To increase the penetration depth of UL bio-imaging *in vivo*, a high excitation power density is required. Paradoxically, further increasing the laser power may cause marked overheating effects, which can lead to possible scalding of animal tissue upon continuous irradiation^8^,^9^. Moreover, infrared solar photons with wavelength greater than 1 μm are unabsorbed by solar cells working in the visible and near-infrared regions, thus UL materials provide a solving method by turning infrared solar photons into visible photons, leading to increased photoelectric conversion efficiency of the solar cells in theory^11^,^12^. However, sun-light is usually not sufficiently strong to activate the UC process. To solve this type of practical problem, research on highly efficient UL materials induced by low excitation power is an urgent task.

The Yb^3+^-Er^3+^ codoped UC system has been most commonly used to obtain efficient UL materials, mainly owing to the large absorption cross section of approximately 980 nm in Yb^3+^, the relatively long lifetime energy levels in Er^3+^ and the good energy level match between these two types of RE ions^13^,^14^,^15^,^16^,^17^,^18^,^19^. Herein, we conducted a series of research on Yb^3+^, Er^3+^ codoped materials and found that one type of UL material, Ba_5_Gd_8_Zn_4_O_21_:Yb^3+^, Er^3+^, could realize bright UC emissions even stronger than β-NaYF_4_:Yb^3+^, Er^3+^ when the excitation power is not high, and its UC optical properties are detailed in this paper.

More interestingly, by increasing the doping concentrations of Ba_5_Gd_8_Zn_4_O_21_:Yb^3+^, Er^3+^ samples, the red emission intensity originating from ^4^F_9/2_ in Er^3+^ increased dramatically, and the red to green emission power ratio went up from lower than 1 to approximately 10. We have noticed that this dynamic change regularity exists not only in Ba_5_Gd_8_Zn_4_O_21_:Yb^3+^, Er^3+^, but also in many other Yb^3+^, Er^3+^ co-doped UL materials such as β-NaYF_4_, NaGdF_4,_ Y_2_O_3_, BaIn_6_Y_2_O_13_, BaGd_2_ZnO_5_, and Y_2_Sn_2_O_7_^13^,^14^,^15^,^16^,^17^,^18^. Considering this fact, it is most likely that in the Yb^3+^-Er^3+^ co-doped UC system the red emission have more efficient UC routes, which can contribute when the doping concentration is sufficiently high; however, this conjecture has not yet been confirmed. In this paper, we successfully proved the existence of CR by establishing the rate equations of the energy levels. In addition, the temporal evolutions of UC emissions from a series of Ba_5_Gd_8_Zn_4_O_21_:Yb^3+^, Er^3+^ samples were recorded and the experimental results can also explained by the CR process.

## Results and Discussion

### Crystal Structure

**Figure 1:**
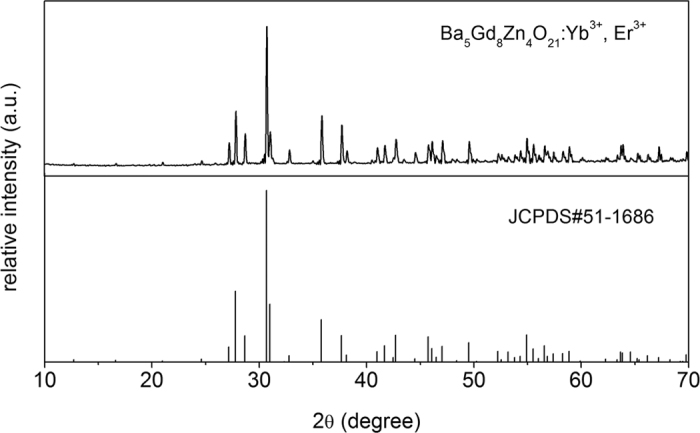
The X-ray powder diffraction. The XRD pattern of the as-synthesized Ba_5_Gd_8_Zn_4_O_21_:Yb^3+^, Er^3+^.

Figure 1 shows the XRD pattern of the as-synthesized Ba_5_Gd_8_Zn_4_O_21_:Yb^3+^, Er^3+^. It can be observed that the diffraction peaks of the sample coincide well with the standard data on Ba_5_Gd_8_Zn_4_O_21_ (JCPDS NO. 51–1686) and no extra peaks from any impurities are seen, which indicates that the dopants Yb^3+^ and Er^3+^ completely enter the lattice of the host. The crystalline structure is tetragonal with space group I4/m.

### Spectroscopic Characterizations and UC Power Efficiency

**Figure 2:**
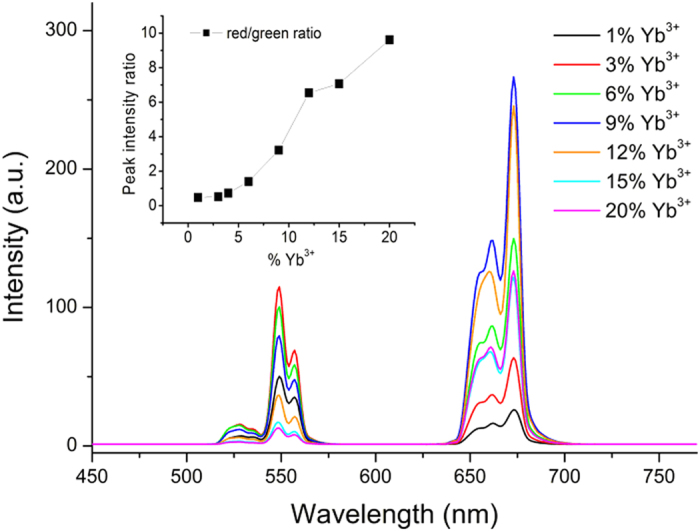
PL upconversion spectra of the sample. UC spectra of seven selected Ba_5_Gd_8_Zn_4_O_21_:x% Yb^3+^, 1% Er^3+^ (x = 1, 3, 6, 9, 12, 15, 20) samples under the 971 nm CW laser excitation (18.6 W/cm^2^), the inset presents the red to green emission intensity ratio of the samples.

The UC optical properties depend on dopant concentrations. Under 971 nm CW laser excitation at r oom temperature, the selected Ba_5_Gd_8_Zn_4_O_21_:x% Yb^3+^, 1% Er^3+^ (x = 1, 3, 6, 9, 12, 15, 20) samples yield green to red emission. In Fig. 2, the spectra exhibit three broad emission bands centered at 548 nm, 557 nm and 672 nm, which are ascribed to the ^2^H_11/2_→^4^I_15/2_, ^4^S_3/2_→^4^I_15/2_ and ^4^F_9/2_→^4^I_15/2_ energy transitions in Er^3+^, respectively. The inset of Fig. 2 shows the red to green UL intensity ratio for the seven selected samples. By increasing the Yb^3+^ concentration and fixing the Er^3+^ concentration to 1%, the red to green emission intensity ratio increases strongly and reaches 0.47, 0.51, 0.72, 1.40, 3.22, 6.54, 7.07, and 9.61 for the samples doped with 1%, 3%, 6%, 9%, 12%, 15%, and 20% Yb^3+^ respectively, and there exists a similar rule in samples with a fixed Yb^3+^ concentration: the red emission intensity grows much faster and higher than that of the green emission by increasing Er^3+^ concentration, as shown in Figure S1. The dependence of UC optical properties on the doping concentration indicates that the red emission level has a more efficient means to be filled than the green emission levels especially in doped samples with Yb^3+^ or Er^3+^ concentration.

**Figure 3:**
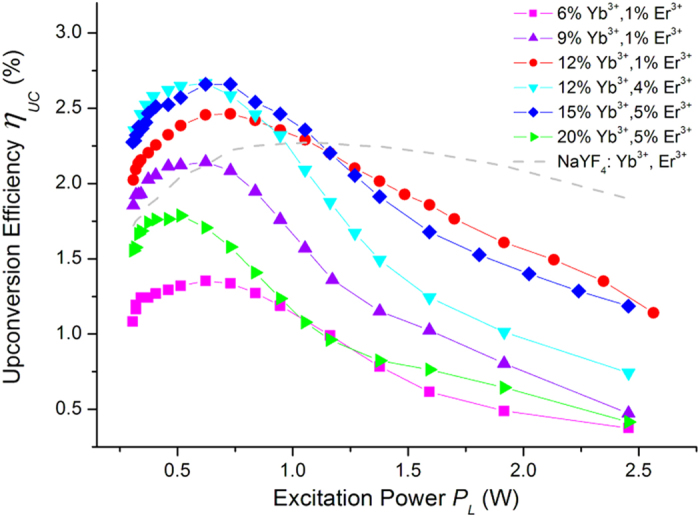
UC power efficiency test. UC power efficiency of several Ba_5_Gd_8_Zn_4_O_21_:Yb^3+^, Er^3+^ phosphors under different excitation power, the UC power efficiency of commercial NaYF_4_:Yb^3+^, Er^3+^ phosphor is also provided as a contrast.

The UC power efficiencies of Ba_5_Gd_8_Zn_4_O_21_:Yb^3+^, Er^3+^ samples, including one commercial NaYF_4_:Yb^3+^, Er^3+^ phosphor as a contrast, were measured at room temperature under 971 nm excitation. As shown in Fig. 3, the UC power efficiency exhibited a similar changing trend: increasing under low excitation power up to ~0.7 W and then decreasing at higher excitation power. A maximum UC power efficiency of 2.7% was obtained with Ba_5_Gd_8_Zn_4_O_21_:12% Yb^3+^, 4% Er^3+^ and Ba_5_Gd_8_Zn_4_O_21_:15% Yb^3+^, 5% Er^3+^ under 0.67 W excitation. It is worth noting that the Ba_5_Gd_8_Zn_4_O_21_:Yb^3+^, Er^3+^ sample has a higher UC power efficiency than the commercial β-NaYF_4_:Yb^3+^, Er^3+^ phosphor when excitation power is low, despite the fact that the β-NaYF_4_ host lattice doped with RE ions is regarded as a high efficiency phosphor and is currently the most widely used. For the emission power of each Ba_5_Gd_8_Zn_4_O_21_:Yb^3+^, Er^3+^ sample please refer to Table S1.

**Figure 4:**
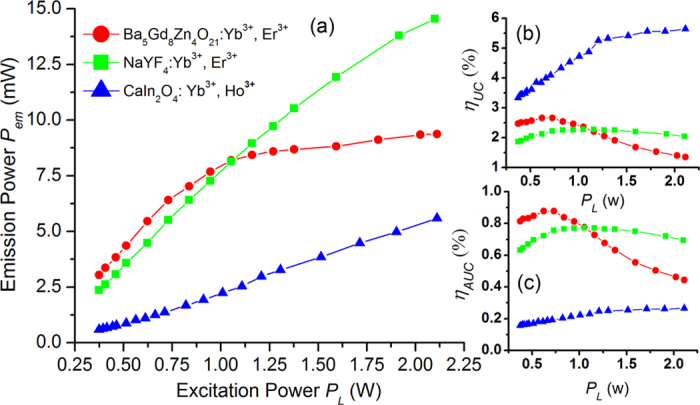
Comparison among three types of UC materials. The comparison of (**a**) emission power, (**b**) UC power efficiency and (**c**) actual UC power efficiency among three types of UL materials: Ba_5_Gd_8_Zn_4_O_21_:15% Yb^3+^, 5% Er^3+^ (red line), CaIn_2_O_4_:10% Yb^3+^, 0.5% Ho^3+^ (green line) and β-NaYF_4_:18% Yb^3+^, 2% Er^3+^ (blue line). The excitation source is a 971 nm power-controllable diode laser.

Many reports have regarded high UC efficiency as a measure of UC materials with strong luminescence^16^,^20^,^21^. Generally, UC efficiency takes three forms: actual UC power efficiency (UC emission power/excitation power), UC power efficiency (UC emission power/absorbed excitation power), and UC quantum efficiency (UC emission quantum numbers/absorbed excitation quantum numbers). In our previous research, we found that the absorptivity to laser radiation varies greatly among different types of phosphors. For example, the phosphor CaIn_2_O_4_:Yb^3+^, Ho^3+^ has a higher UC power efficiency (approximately 5%) than Ba_5_Gd_8_Zn_4_O_21_:Yb^3+^, Er^3+ ^20, whereas the emission power of CaIn_2_O_4_: Yb^3+^, Ho^3+^ is much lower than that of Ba_5_Gd_8_Zn_4_O_21_:Yb^3+^, Er^3+^ under the equal power excitation, as shown in Fig. 4 (a,b). The CaIn_2_O_4_: Yb^3+^, Ho^3+^ is more efficient because of its much lower absorptivity to 971 nm laser radiation than Ba_5_Gd_8_Zn_4_O_21_:Yb^3+^, Er^3+^; the absorptivity of Ba_5_Gd_8_Zn_4_O_21_:Yb^3+^, Er^3+^ is approximately 30%, and that for CaIn_2_O_4_:Yb^3+^, Ho^3+^ is only approximately 5%. Obviously, CaIn_2_O_4_:Yb^3+^, Ho^3+^ does not have high emission power, and judging only from the UC efficiency will mislead us in finding the appropriate UL materials. Actually, how much excitation power could be converted into UC emission power is our focus, which has nothing to do with the absorptivity of the material. The core intent here is to find phosphors with high emission power in low excitation power regions. Thus, we deem it necessary to also note the actual UC power efficiency for UC material studies, which considers only the relationship between the emission power and the pumping power. The results in Fig. 4 (c) indicate that the highest actual UC power efficiency for Ba_5_Gd_8_Zn_4_O_21_:Yb^3+^, Er^3+^ is 0.87% when the excitation power is approximately 0.67 W, and it can produce stronger UL than the other two materials in Fig. 4. More details of the actual UC power efficiency for Ba_5_Gd_8_Zn_4_O_21_:Yb^3+^, Er^3+^ can be seen in Figure S2.

**Figure 5:**
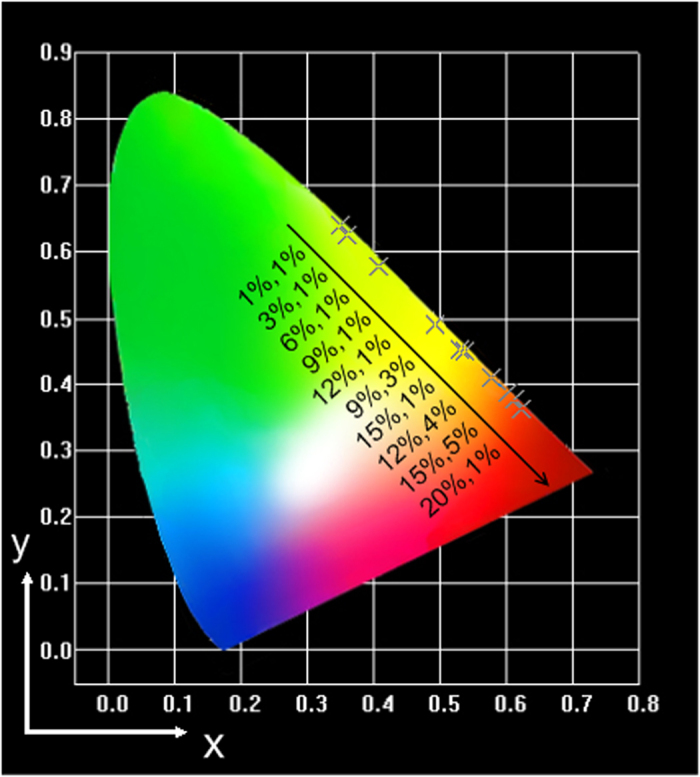
The CIE chromaticity diagram. CIE chromaticity diagram and x, y color coordinates (cross mark) of several Ba_5_Gd_8_Zn_4_O_21_:Yb^3+^, Er^3+^ phosphors, the percentages of each group represent the concentration of Yb^3+^, Er^3+^, respectively.

The *x* and *y* color coordinates associated with the emission of the Ba_5_Gd_8_Zn_4_O_21_:Yb^3+^, Er^3+^ phosphor family are positioned on a CIE *x*, *y* chromaticity diagram in Fig. 5; the excitation power is 0.65 W. It can be seen that when the Yb^3+^ or Er^3+^ concentration reaches a higher value, the color of the UC emission turns from yellowish green to reddish orange, which indicates that the Ba_5_Gd_8_Zn_4_O_21_:Yb^3+^, Er^3+^ phosphor has excellent color-tunable properties.

### UC Mechanisms

The pump power dependence of the green and red emissions was investigated at room temperature under 971 nm excitation. Notably, under high excitation power, the slope *l* decreased to less than 1 as a result of the competition between linear decay and UC processes for the depletion of the intermediate excited states according to M. Pollnau’s report and our previous work^21^,^22^. Please refer to the Supporting Documents for more details.

Ground state absorption (GSA), subsequent UC by excited state absorption (ESA) and energy transfer (ET) UC were the main UC mechanisms in the population for excited states, additional processes such as CR between two doped ions or an avalanche process may occur as well^23^. The GSA/ESA process involves a single ion, so it is the main possible UC process that occurs in materials with low dopant concentrations, whereas ET involves two neighboring ions and will be dominant in the materials with high doping concentrations by shortening the average distance between dopant ions and enhancing the interionic interaction.

**Figure 6:**
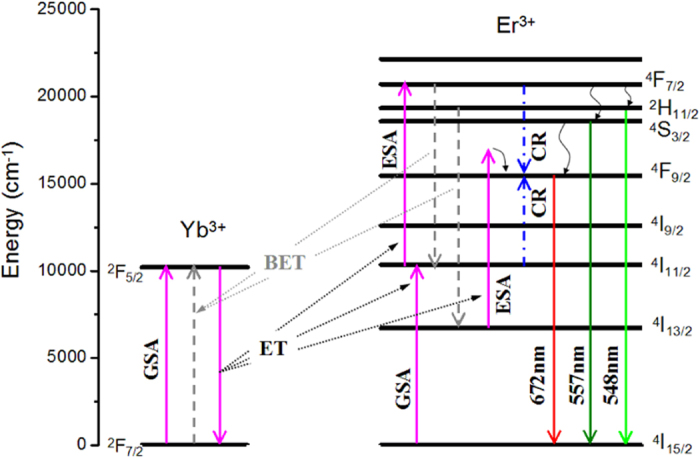
The energy level diagram. Energy level diagram of Yb^3+^ and Er^3+^ and the proposed UC mechanism in Ba_5_Gd_8_Zn_4_O_21_:Yb^3+^, Er^3+^.

Figure 6 presents the energy level diagrams of Yb^3+^ and Er^3+^ ions^24^,^25^, and the proposed UC routes in Ba_5_Gd_8_Zn_4_O_21_:Yb^3+^, Er^3+^ are also listed. Because Yb^3+^ ions have a larger absorption cross-section at the NIR region and a high doping concentration, the ET process from Yb^3+^ to Er^3+^ makes prominent contributions to the population of the emission energy levels. The UC route for the green emission is simple and we do not discuss it here. For the red emission originating in the energy level ^4^F_9/2_, most of the relevant literature asserted that are two different routes. One route is non-radiative relaxation from the upper level ^4^S_3/2_ to ^4^F_9/2_, and the second route is to populate the long-lived ^4^I_13/2_ level via non-radiative relaxation of ^4^I_11/2_ → ^4^I_13/2_^26^,^27^, followed by an ET process: ^2^F_5/2_ (Yb^3+^) + ^4^I_13/2_ (Er^3+^) → ^2^F_7/2_ (Yb^3+^) + ^4^F_9/2_ (Er^3+^) or an ESA process: photon + ^4^I_13/2_ (Er^3+^) → ^4^I_15/2_(Er^3+^) + ^4^F_9/2_ (Er^3+^). In fact, the energy gap between ^4^I_13/2_ and ^4^F_9/2_ is approximately 8700 cm^−1^, and the energy of one 971 nm photon is approximately 10300 cm^−1^, which is much higher than the energy gap; therefore, the ET and ESA processes from the ^4^I_13/2_ level will be restricted owing to the absence of the energy match condition. In addition, the positions of the energy level for Yb^3+^ and Er^3+^ ions in different hosts remain almost the same because of the shielding effect of their outer closed 5 s^2^ and 5 p^6^ shields.

As noted above, CR occurs between two ions and requires a good coincidence between the energy gaps involved in this process. From the energy level diagram of Er^3+^, we found that the energy gap between ^4^F_7/2_ and ^4^F_9/2_ (approximately 5200 cm^−1^) matches well with the energy gap between ^4^F_9/2_ and ^4^I_11/2_ (approximately 5100 cm^−1^)^24^. Moreover, both ^4^F_7/2_ and ^4^I_11/2_ could be directly filled by the ET process, which guarantees major populations in the two energy levels. In fact, Liao reported that this cross-relaxation process may result in an enhanced population of ^4^F_9/2_^17^. Unfortunately, they have not provided a more detailed explanation.

As shown in Fig. 6, the CR process between Er^3+^ ions is expressed as follows: ^4^F_7/2_ (Er^3+^) + ^4^I_11/2_ (Er^3+^) → ^4^F_9/2_ (Er^3+^) + ^4^F_9/2_ (Er^3+^); the doubling of the ions in ^4^F_9/2_ offers a cogent reason for the strong red emission. As seen in the previous section, when Yb^3+^ concentration is lower, the absorbed excitation power is too low to maintain plentiful populations in ^4^I_11/2_ and ^4^F_7/2_, and the ions in ^4^F_7/2_ will relax to the green emission levels preferentially. The CR process only negligibly populates the red emission level. Thus, the green emission is stronger than the red emissions owing to the low Yb^3+^ concentration. In contrast, because the Yb^3+^ concentration increases to a higher value, the ET process from Yb^3+^ to Er^3+^ becomes more active to populate the excited levels, which guarantees a greater contribution made by the three red level population routes, especially doubling the number of ions in ^4^F_7/2_ caused by CR processes. In view of this fact, the red emission increased dramatically with the Yb^3+^ concentration. For the samples with fixed Yb^3+^ concentration, high Er^3+^ concentration will shorten the average distance between Er^3+^ ions and enhance the CR process; thus, the increasing intensity of red emission rises with increasing Er^3+^ concentration can be explained.

### The Rate Equation Model

**Figure 7:**
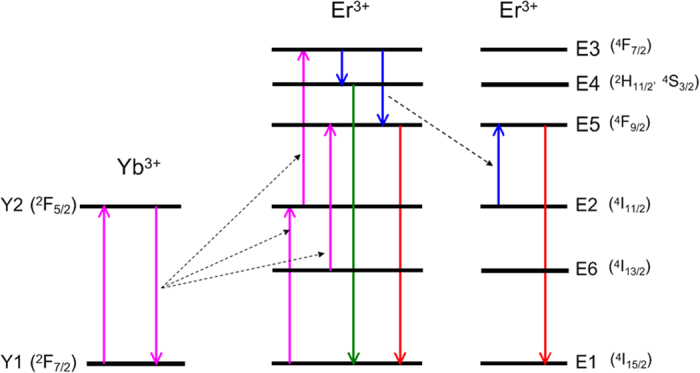
The energy level diagram for rate equation analysis. The simplified Yb^3+^-Er^3+^ UC system in Ba_5_Gd_8_Zn_4_O_21_:Yb^3+^, Er^3+^.

To prove the existence of the CR process in Er^3+^ ions, we have simplified the Yb^3+^-Er^3+^ UC system in Ba_5_Gd_8_Zn_4_O_21_:Yb^3+^, Er^3+^, as shown in Fig. 7, and listed the following assumptions to clarify the rate equation concisely:

1. UC steps between sequential excited states take place through ET, GSA or ESA, the main population route in excited states of Er^3+^ is ET from Yb^3+^ ions, which has been demonstrated^22^,^28^.
2. Radiative transition is the main depopulation route in green and red emission levels E4 and E5.

Initially the rate equations describing the excitation mechanisms in this system can be written as^21^,^22^,^28^:


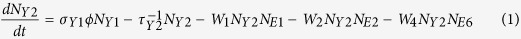



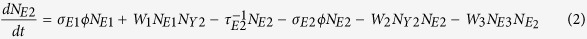



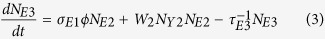



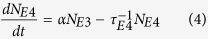


where *N*_*i*_ is the population in energy level *i*, *σ*_*i*_ is the absorption cross section of state *i* at the pump wavelength, *ϕ* is the luminous flux of the pump light, *τ*_*i*_ is the lifetime of state *i* (the excited states *i* decay with rate constants *τ*_*i*_^*−1*^), and *α* is the relaxation rate from E3 to E4. The energy transfers from state *i* to state *j* are described by the factor W_k_*N*_*i*_*N*_*j*_, in which the constant W_k_ represents the energy transfer rate from state *i* to state *j*.

According to the assumptions, the rate equations above could then be rewritten as


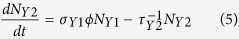



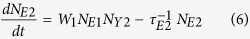



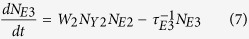



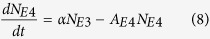


where *A*_*i*_ represents the radiative transition probabilities of state *i*.

For the rate equation of red emission level E5, there are three limiting cases corresponding to three means of populating E5:

(1) If red emission level E5 is populated only by the proposed CR process between states E2 and E3 in Er^3+^ ions, the rate equation of red emission level E5 is


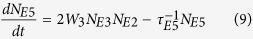


which can be rewritten as:


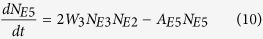


Under steady-state excitation, Equation 5, Equation 6, Equation 8 and Equation 10 yield:


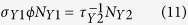



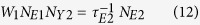



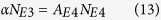



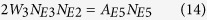


In this situation, the red to green emission intensity ratio *R* can be expressed as:


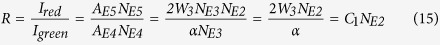


where C_i_ represent a constant. If the Yb^3+^ concentration is fixed, then both *N*_*Y1*_ and *N*_*Y2*_ are constants based on Equation 11, and it follows from Equation 15 and Equation 12 that


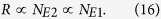


If the Er^3+^ concentration is fixed such that *N*_*E1*_ is constant, it follows from Equation 15, Equation 12 and Equation 11 that


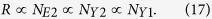


As a result, the ground state populations *N*_*E1*_ and *N*_*Y1*_are proportional to the dopant concentration, and based on Equation 16 and Equation 17, we could reach the conclusion that the red to green emission intensity ratio *R* is proportional to the dopant concentration of Yb^3+^ and Er^3+^. This result agrees with experimental data.

(2) If the red emission level E5 is populated only by the ET process from Y2 to E6, the rate equations of E5 and E6 are


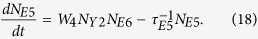



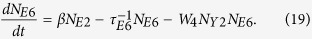


both of which could be rewritten as


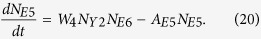



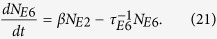


where *β* represents the relaxation rate from E2 to E6.

Under steady-state excitation, Equation 7, Equation 20 and Equation 21 yield


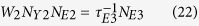



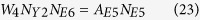



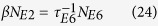


*R* can then be expressed as


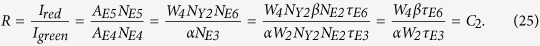


In this situation, R is a constant and the red to green emission intensity ratio will not vary with Yb^3+^ or Er^3+^ concentration, which contradicts the experimental results.

(3) If red emission level E5 is populated only by the relaxation from E3, the rate equation of E5 is


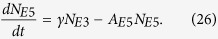


where γ represents the relaxation rate from E3 to E5, under steady-state excitation, this yields


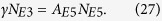


and *R* can be expressed as:


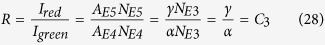


Obviously, R will not vary with Yb^3+^ or Er^3+^ concentration, which contradicts the experimental results.

According to the rate equation analysis, the CR process as the third means to generate the red emission is reasonable and necessary.

### Lifetime Measurements

**Figure 8:**
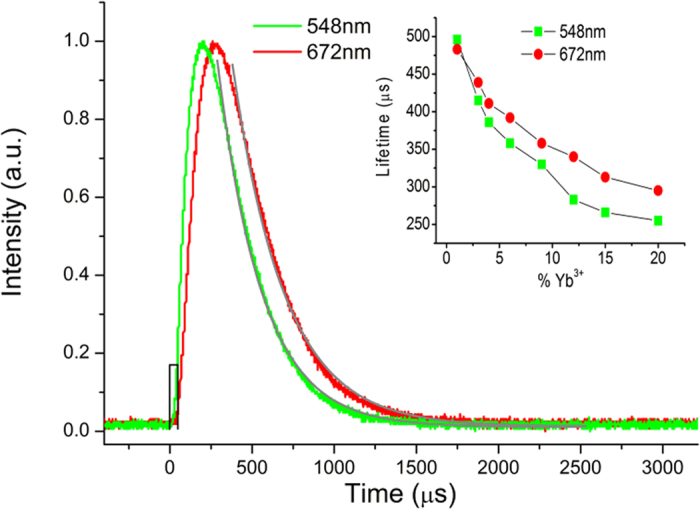
The lifetime measurements for Ba_5_Gd_8_Zn_4_O_21_:Yb^3+^, Er^3+^. The temporal evolutions of the green emission at 548 nm and red emission at 672 nm under 971 nm pulsed laser excitation and the fitted curve of Ba_5_Gd_8_Zn_4_O_21_:15% Yb^3+^, 1% Er^3+^. The inset presents the calculated lifetime for both the green emission and the red emission of the selected Ba_5_Gd_8_Zn_4_O_21_:x% Yb^3+^, 1% Er^3+^ (x = 1, 3, 6, 9, 12, 15, 20) samples under the 971 nm pulsed laser excitation.

The temporal evolutions of ^2^H_11/2_→^4^I_15/2_ (green) and ^4^F_9/2_→^4^I_15/2_ (red) transitions of Er^3+^ ion will also be helpful in understanding the UC processes, as shown in Fig. 8. In fact, all decay curves can be fitted by solving the rate equations. In view of the complexity, we can obtain the lifetime of red and green emission by fitting the decay parts of the lifetime curve with a single exponential function. According to Equation 10, after the green level stops being populated, the change in the population of the green level is written as


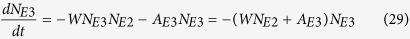


This could be rewritten as


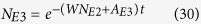


In the same way, after the red level stops being populated, the population of the red level can be written as


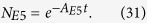


Thus, the emission decay curves of the green and red emission can be well fitted with a single exponential function. The inset presents the calculated lifetime for both the green and red emissions of different samples. Before specifically analyze on the temporal evolutions, we summarize the key findings in the lifetime measurements:

1. For both green and red emissions, there exist a delayed rise and a decay in the emission intensity after the end of the excitation pulse.
2. The red emission, in contrast, has a longer rise time than the green emission.
3. The lifetime of both green and red emissions decreases with increasing Yb^3+^ doping concentration.
4. The green and red emissions have similar lifetime in the sample doped with a low concentration of Yb^3+^, but once the Yb^3+^ concentration reaches to a higher value, the lifetime of green emission decreases more quickly than the lifetime of red emission.

Observation (1) clearly indicates the existence of an energy transfer process. The ET process from Yb^3+^ to Er^3+^ and the proposed CR from Er^3+^ to Er^3+^ can still happen when the excitation is complete, which leads to a delayed rise of the emission intensity. Observation (2) suggests that the red emission level ^4^F_9/2_ requires longer time than the green emission level to be populated. According to the UC mechanisms described above, CR occurs after the population of Er^3+^ energy levels, which means that the red emission caused by CR requires additional time after ET from Yb^3+^ to Er^3+^. The green emission does not require such a long time owing to the lack of additional ET or CR processes required. Observation (3) can be explained by the back energy transfer (BET) from Er^3+^ to Yb^3+ ^19. Once the excitation is complete, the high energy state ^4^F_7/2_ and ^2^H_11/2_ in Er^3+^ can transfer energy to Yb^3+^ ions in the ground state, and the higher the Yb^3+^ concentration is, the stronger BET is likely to take place; the lifetime of both green and red emissions will then decrease with increasing Yb^3+^ concentration. The related data can be seen in Figure S5. Observation (4) once again proved the existence of the proposed CR process. According to Equation 30, the lifetime *τ*_*G*_ of green emission can be written as


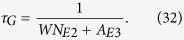


The lifetime *τ*_*R*_ of red emission can be written as:


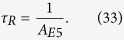


The lifetime *τ*_*G*_ of green emission will decrease with increasing Yb^3+^ concentration. However, the lifetime *τ*_*R*_ of red emission does not change with increasing Yb^3+^ concentration. Thus, the lifetime of green emission decreases more quickly than the lifetime of red emission when the Yb^3+^ concentration increases.

The key point of the CR process is the multiplier effect to the population of ^4^F_9/2_ in Er^3+^, which is similar to the photon avalanche (PA) as shown in Figure S4(a). However, PA suffered from some drawbacks^29^,^30^,^31^,^32^. One is that the pump wavelength does not match the energy gap between the ground state and the intermediate excited level, possibly leading to the intermediate excited level being populated only by weak GSA initially. Another disadvantage is that such a process always requires high pump powers to reach the threshold condition, below which the UC luminescence intensity is weak. Moreover, PA has a longer rise time because many looping cycles are required to achieve avalanche. Compared with the PA process, the proposed CR process combines the advantages of efficient ET in a Yb^3+^, Er^3+^ co-doped system with the multiplier effect to populate the red emission level. Especially when the doping concentration is sufficiently high, frequent ET from Yb^3+^ to Er^3+^ guarantees a substantial population in the excited states of Er^3+^, promoting CR between Er^3+^ ions. Moreover, the multiplier effect caused by PA takes place in the intermediate excited level, whereas in the CR process the multiplier effect benefits the UC emission level directly. More significantly, the proposed CR process does not require a pumping power threshold condition to have an immediate effect on the UC emission.

## Conclusion

UC properties of the oxide material Ba_5_Gd_8_Zn_4_O_21_:Yb^3+^, Er^3+^ were investigated as a function of Yb^3+^ and Er^3+^ dopant concentration under 971 nm excitation. The phosphors exhibit strong UC emissions, including a green emission band at 548 nm and a predominant red emission band at 672 nm. Based on the power dependence studies, we provided a model for the UC mechanisms involved in these materials. The UC efficiency testing indicated that Ba_5_Gd_8_Zn_4_O_21_:Yb^3+^, Er^3+^ had a maximum UC power efficiency of 2.7% under low power excitation, and is thus more efficient than the commercial NaYF_4_:Yb^3+^, Er^3+^ phosphor. Together with the colour-turnable property and the stable nature of oxide, Ba_5_Gd_8_Zn_4_O_21_:Yb^3+^, Er^3+^ has the potential to be an ideal UC phosphor.

In particular, we demonstrated that the CR process occurred between two Er^3+^ ions through rate equations; based on this fact, the strong red emission appearing in different Yb^3+^, Er^3+^ codoped UL materials can be fully explained for the first time, and the temporal evolution of the green and red emission improved this idea.

## Methods

### Compounds Synthesis

The compound Ba_5_Gd_8−x−y_Yb_x_Er_y_Zn_4_O_21_ was prepared by the typical high-temperature solid state method, in which Yb^3+^ and Er^3+^ were added as a sensitizer and activator, respectively. The stoichiometric amount of starting materials BaCO_3_ (Alfa Aesar, 99.99%), ZnO (Fisher Scientific, 99.5%), Gd_2_O_3_ (Alfa Aesar, 99.99%), Yb_2_O_3_ (Alfa Aesar, 99.99%) and Er_2_O_3_ (Alfa Aesar, 99.99%) were thoroughly mixed, ground together in an agate mortar and filled into an aluminum oxide crucible. The mixture was then sintered at 1400 °C for 3 h, followed by cooling to room temperature spontaneously. After a second grinding stage we obtained the final product of Ba_5_Gd_8_Zn_4_O_21_:Yb^3+^, Er^3+^ power.

### XRD Characteristic

X-ray powder diffraction patterns were measured by a Bruker D8 advance X-ray diffractometer (Bruker Optics, Germany) with Cu Kα radiation in the range 10° ≤ 2θ ≤ 70°. The UC luminescence spectra were recorded on an Andor SR-500 i spectrometer (Andor Technology Co., UK) equipped with a Hamamatsu R928 photomultiplier. A power-controllable 971 nm laser diode (BWT Beijing Ltd., China) was used as the excitation source, which can produce both continuous-wave and pulsed laser radiation. For the UC luminescence lifetime measurements, the UC emissions under the 971 nm pulse laser (pulse width = 50 μs) were passed through the Andor SR-500 i spectrometer and detected by the Hamamatsu R928 photomultiplier connected to a 1 GHz Tektronix digital oscilloscope.

### Optical Measurements

The excitation source used for the UC efficiency measurement was a 971 nm controlled temperature CW semiconductor diode laser (BWT Beijing Ltd., China) with P_max_ = 3 W. The copper sample holder in the middle of the integrating sphere was excited by the diode laser. After multiple reflections in the integrating sphere, the emitted UC light passed through an optical fiber and was analyzed with a spectrometer (380–800 nm) and a relative luminance meter. The laser output power P_L_ under different currents was measured by a LP-3A laser power meter (Physcience Opto-Electronics Co., China). After initial calibration of the setup, the efficiency was determined in two steps. For the first measurement, the copper sample holder in the integrating sphere was left empty, and the laser spectrum was obtained by a spectrometer. From this step, we obtained the integrated intensity I_L_ over the range 950–1000 nm. For the second measurement, the copper sample holder in the integrating sphere was filled with the sample. From this step, we obtained the UC emission power P_em_ in the range 380–800 nm and the integrated intensity I_unabs_ in the range 950–1000 nm. Finally the UC power efficiency η_UC_ was calculated as the ratio of the luminescence power P_em_ emitted by sample over the power P_abs_ absorbed in the infrared range 950–1000 nm:


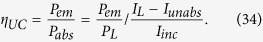


and the actual UC power efficiency η_AUC_ is defined as:


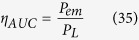


which represents the ratio of the UC emission power to the laser output power. We can calculate the excitation density based on the following formula: the excitation density = excitation power/laser spot area. We measured the excitation power by the laser power meter and the laser spot area could be calculated by the formula *S* = *πR*^*2*^sin*θ*, where *R* and *θ* represent the radius of the laser spot and the angle between the sample surface and the fiber end, respectively. R equals the distance between the sample surface and the fiber end multiplied by 0.22 (provided by the manufacturer), and *θ* is 45°.

## Additional Information

**How to cite this article**: Mi, C. *et al*. Efficient upconversion luminescence from Ba_5_Gd_8_Zn_4_O_21_:Yb^3+^, Er^3+^ based on a demonstrated cross-relaxation process. *Sci. Rep*. **6**, 22545; doi: 10.1038/srep22545 (2016).

## Supplementary Material

Supplementary Information
