# Supplementary material for: Efficient upconversion luminescence from Ba5Gd8Zn4O21:Yb3+, Er3+ based on a demonstrated cross-relaxation process
Source: Sci Rep. 2016 Mar 2;6:22545. doi: 10.1038/srep22545 (PMC4773834; doi:10.1038/srep22545)
Supplement: Supplementary Information [file srep22545-s1.doc]

Supporting Information

**Efficient upconversion luminescence from Ba_5_Gd_8_Zn_4_O_21_:Yb^3+^, Er^3+^** **based on a demonstrated cross-relaxation process**

***Chao Mi, Jianhong Wu, Yanmin Yang,^*^ Boning Han, and Jun Wei***


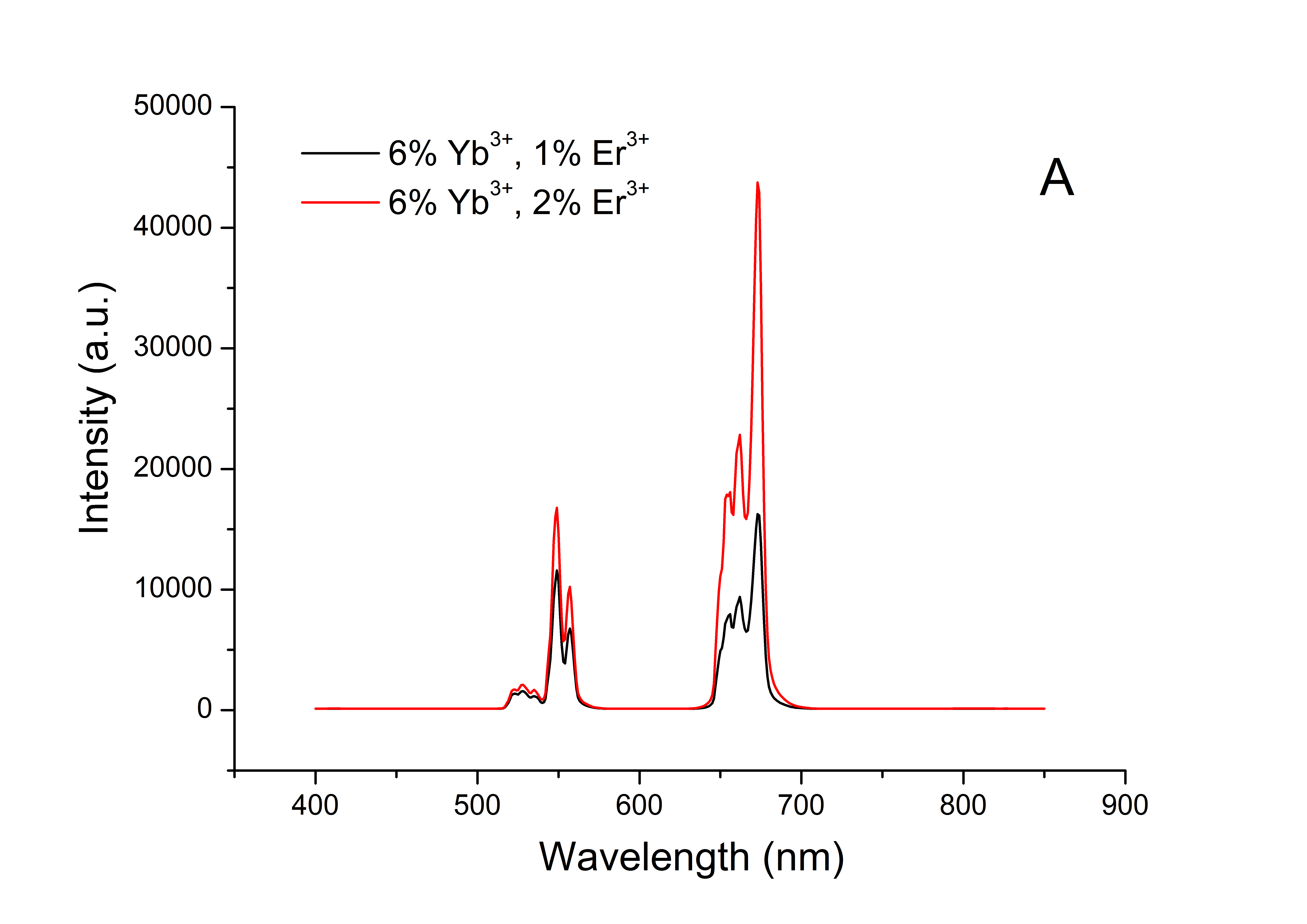

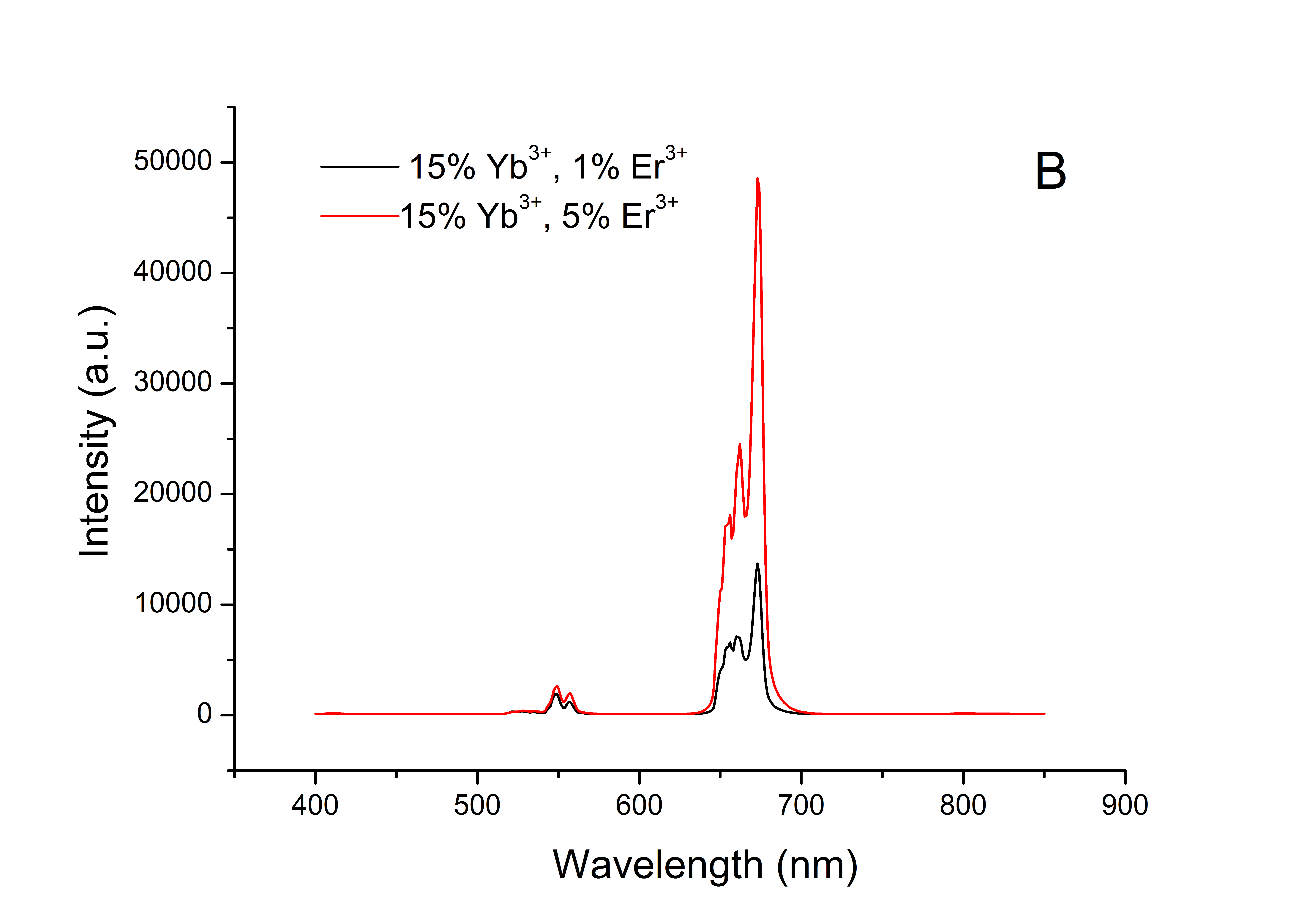


**Figure S1.** **PL upconversion spectra**. UC spectra of two groups of Ba_5_Gd_8_Zn_4_O_21_: Yb^3+^, Er^3+^ samples under the 971nm CW laser excitation, (A) Ba_5_Gd_8_Zn_4_O_21_: 6% Yb^3+^, y% Er^3+^ (y=1, 2), (B) Ba_5_Gd_8_Zn_4_O_21_: 15% Yb^3+^, y% Er^3+^ (y=1, 5). It can be seen that the red emission increases more significantly than green emission by increasing Er^3+^ concentration.

**Table S1.** The green emission power *P_green_*, red emission power *P_red_* and total emission power *P* of the as-prepared Ba_5_Gd_8_Zn_4_O_21_:Yb^3+^, Er^3+^ samples. The excitation power of the 971 nm laser is 0.73 W.

| Ba_5_Gd_8_Zn_4_O_21_:  Yb^3+^, Er^3+^ | *P*_green_ (mW) | *P*_red_ (mW) | *P* (mW) |
| --- | --- | --- | --- |
| 3%, 1% | 1.04666 | 1.40864 | 2.4553 |
| 6%, 1% | 0.74011 | 1.99032 | 2.73043 |
| 9%, 1% | 0.57401 | 3.53436 | 4.10837 |
| 9%, 3% | 0.30178 | 2.95066 | 3.25244 |
| 12%, 1% | 0.62861 | 5.12149 | 5.7501 |
| 12%, 4% | 0.3069 | 5.35223 | 5.65913 |
| 15%, 1% | 0.26038 | 3.46877 | 3.72915 |
| 15%, 5% | 0.30987 | 6.09231 | 6.40218 |
| 20%, 1% | 0.29171 | 5.51903 | 5.81074 |


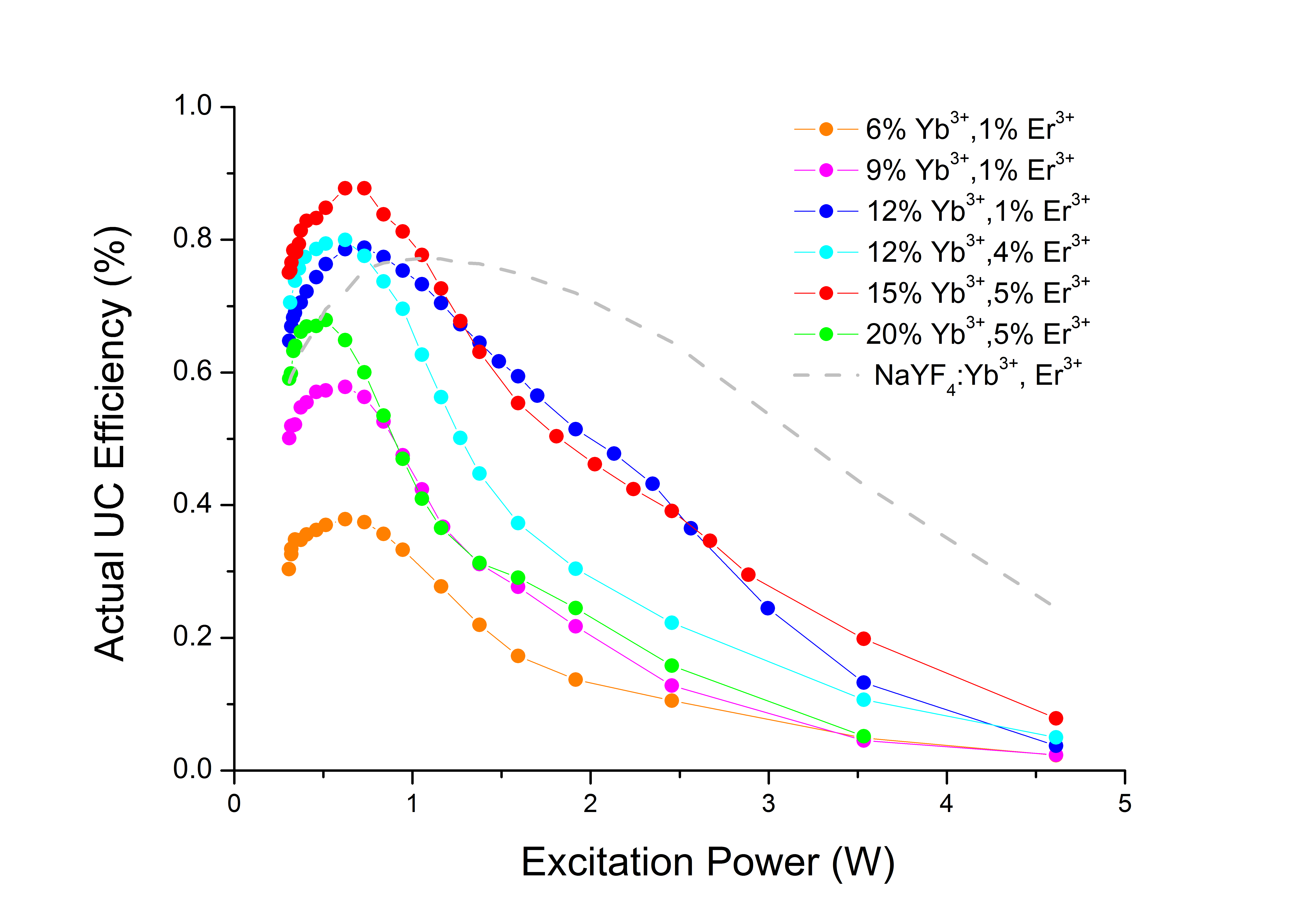


**Figure S2.** **Actual upconversion power efficiency test.** Actual upconversion power efficiency of several Ba_5_Gd_8_Zn_4_O_21_: Yb^3+^, Er^3+^ phosphors under different excitation power, the actual UC power efficiency of commercial NaYF_4_: Yb^3+^, Er^3+^ phosphor is provided as a contrast.


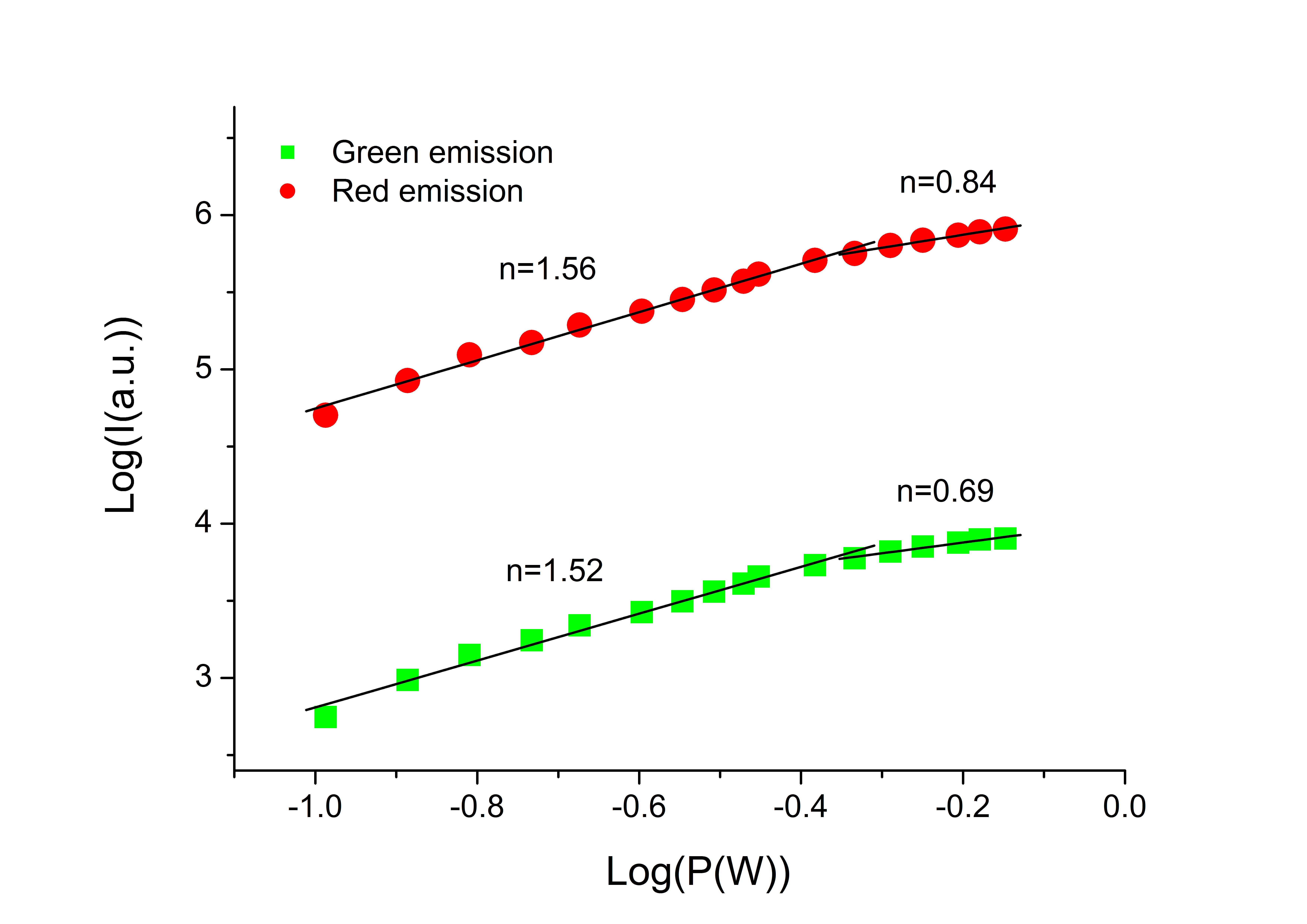


**Figure S3.** **The log-log graph.** The dependence of the upconversion emission intensity on excitation power density for Ba_5_Gd_8_Zn_4_O_21_:Yb^3+^, Er^3+^.


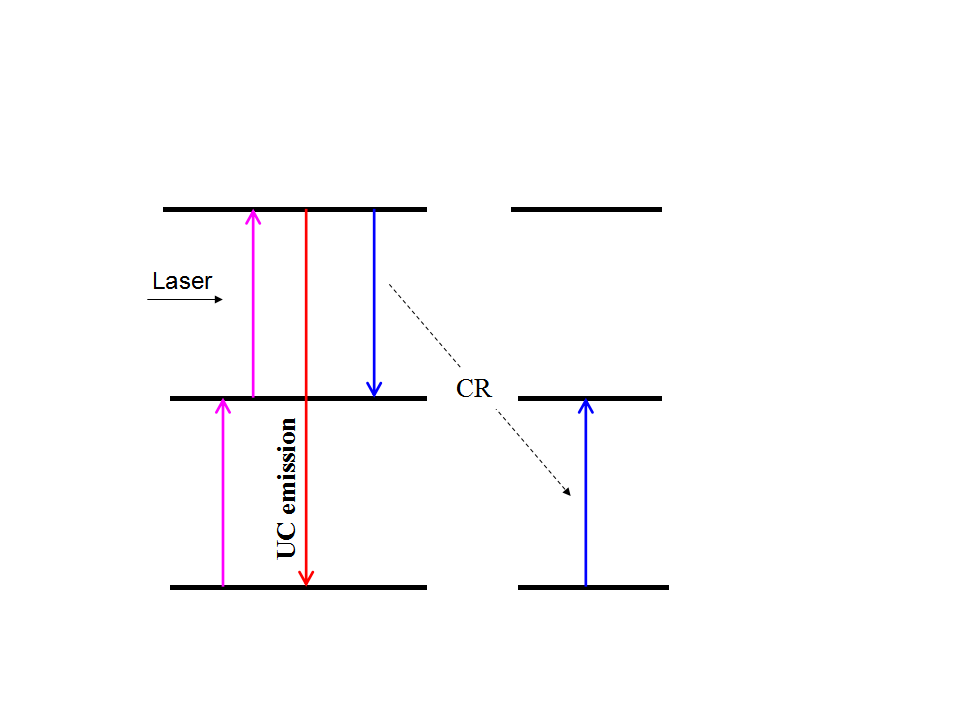


**Figure S4.** **The PA process.** UC mechanism for the simplest PA process.

**Figure S5.** **The emission spectra of Ba_5_Gd_8_Zn_4_O_21_:Yb^3+^, Er^3+^ pumped by a 532 nm laser.** The broad emission band in the spectra has proved the energy transfer from Er^3+^ to Yb^3+^_。_
